# The inhibitory activity of cocoa phenolic extract against pro-inflammatory mediators secretion induced by lipopolysaccharide in RAW 264.7 cells

**DOI:** 10.1186/s40064-016-2138-0

**Published:** 2016-04-28

**Authors:** Yazan Ranneh, Faisal Ali, Mothanna Al-Qubaisi, Norhaizan Mohd Esa, Amin Ismail

**Affiliations:** Department of Nutrition and Dietetics, Faculty of Medicine and Health Sciences, Universiti Putra Malaysia (UPM), 43400 Serdang, Selangor Malaysia; Hematology Department, Faculty of Medicine and Health Sciences, University Hospital, Sana’a University, Sana’a, Yemen; Laboratory of Molecular Biomedicine, Institute of Bioscience, Universiti Putra Malaysia (UPM), 43400 Serdang, Selangor Malaysia

**Keywords:** Cocoa, Polyphenols treatment, Inflammation, 5-Lipoxygenase, Prostaglandin, RAW 264.7 macrophage cells

## Abstract

Cocoa is a rich source of polyphenols that has been traditionally used as the treatment of several types of inflammation related disease. The response to inflammation comprises the consecutive release of mediators and the enlistment of circulating leukocytes, such as macrophages. Currently, Cocoa-derived polyphenolics have shown anti-inflammatory effects in vivo, but the therapeutic benefits in vitro remain unclear. Therefore, in this study, the effect of cocoa polyphenolic extract (CPE) on RAW 264.7 macrophage cells sensitized by lipopolysaccharide as in vitro inflammatory model was investigated. The anti-inflammatory activity of CPE was assessed by measuring its ability to inhibit the pro-inflammatory enzyme 5-lipoxygenase (5-LOX) and the pro-inflammatory mediators prostaglandin E_2_ (PGE_2_), reactive oxygen species (ROS), nitric oxide (NO) and tumor necrosis factor-alpha (TNF-α). The results show that CPE significantly inhibits 5-LOX activity (*p* < 0.01). In addition, CPE dose-dependently suppressed the production of PGE_2_, ROS, NO and TNF-α in RAW 264.7 cells. These data suggest that CPE may be used for the treatment of inflammation and it’s related-diseases.

## Background

Reactive oxygen species (ROS) are naturally and continuously produced as a result of cellular metabolism in all aerobic organisms. Many studies have indicated the deleterious effect of ROS in deteriorating health (Datta et al. 2000; Ali et al. 2013). In addition, a wide range of diseases associated with inflammation are correlated with a high production of ROS (Reuter et al. 2010). During inflammation, respiratory bursts produced by inflammatory cells lead to the increased production and accumulation of ROS at the site of damage (Hussain et al. 2003). Conversely, mitochondrial ROS inhibitors reduce the production of lipopolysaccharide (LPS)-induced IL-6, suggesting the existence of other inhibitions for inflammatory mediators (Edwina and Vishva 2013; Naik and Dixit 2011).

The response to inflammation comprises the consecutive release of mediators and the enlistment of circulating leukocytes, such as macrophages, that become stimulated at the area of inflammation, thereby releasing various types of mediators and cytokines with either pro- or anti-inflammatory actions, such as IL-1β, IL-6, NO, TNF-α and PG (Day 2002; Feldmann et al. 1996). These inflammatory mediators have either pro- or anti- inflammatory actions (Bessis and Boissier 2001). Cytokines incite the chemotactic efflux of monocytes, granulocytes, mast cells and lymphocytes to tissues to support antigen elimination and tissue revival (Eigler et al. 1997; Ershler and Keller 2000). Excessive leakage and stimulation of cells induce tissue damage, resulting in pain and edema (vascular perfusion), which are similar in appearance to inflammation.

TNF-α is an essential factor for the stimulation of the genetic expression of inducible nitric oxide synthase (iNOS) in various cells lines (Wolf et al. 2005). In addition, iNOS is extremely important in macrophages, where its activation results in nitric oxide production (Mac Micking et al. 1997; Vane et al. 1994; Adams et al. 2002), that not only causes organ devastation in some autoimmune and inflammatory diseases but also adjusts various physiological mechanisms, such as vasodilatation (Marletta et al. 1998; Moncada et al. 1991).

During inflammation, the production of fatty acids, including arachidonic acid, which is the main harbinger of fatty acid metabolites, is considerably increased (Kuehl and Egan 1980). Arachidonic acid is first secreted from the cellular membrane by phospholipase enzymes (Burdan et al. 2006) and then transformed by either cyclooxygenase (COX-2) to prostaglandins (PGs) (Pang and Hoult 1997), or by lipoxygenase (LOX) to leukotrienes (LT) (Khanapure et al. 2007). Excessive PGE_2_ produced by COX-2 induces various inflammatory cytokines. On the other hand, the 5-LOX-catalyzed production of LT from plaque cells has been demonstrated to support the inflammatory state in endothelial cells through the flux of leukocytes and the vasoconstriction of arteries (Back 2008).

Because NO, LT and PGE_2_ are the main factors promoting inflammation and pain, the inhibition of the biosynthesis of these inflammatory intermediaries by blocking the TNF-α-NO, PGE_2_-COX2 and 5-LOX-LT pathways, which are the primary pathways responsible for their inflammatory action, is hypothesized to be a promising approach for reducing undesired inflammatory effects. Although corticosteroids and non-steroidal anti-inflammatory drugs (NSAIDs) exert an inhibitory effect on these pathways (Hunskaar and Hole 1987), most NSAIDs exhibit an unwanted side effect on the central nervous, renal, coagulation, cardiovascular and immune systems and the gastrointestinal tract (Rainsford 1999; Mukherjee et al. 2001). Thus, it is very important to decrease the side effects of inflammatory medications by using a different drug or administering the medication in combination with natural products, such as cocoa.

Cocoa is a product of the seeds of the cacao tree (*Theobroma cacao* L.), which is native to the low Andean foothills and the Amazon and Orinoco River basins. It is interesting to note that theobroma means “food of the gods,” as translated from Greek (Keen 2001). Cocoa has been applied for therapeutic purposes to cure several disorders, such as fever, indigestion, angina and heart, liver and lung diseases (Keen 2001; Seligson et al. 1994). Polyphenols, which are widely found in plants, are the primary antioxidative component of cocoa and can be classified into various subclasses, such as flavanols and procyanidins. The intake of chocolate rich in flavonoids by individuals demonstrated a significant decrease in the plasma level of cysteine leukotrienes and prostacyclin (prostaglandin I_2_) (Schramm et al. 2001). As reported by previous studies, cocoa polyphenols exhibit potential health benefits for several chronic diseases, including cardiovascular illness, neurodegenerative disorders and prostate cancer (Kurosawa et al. 2005; Bisson et al. 2008). Many studies on the anti-inflammatory efficacy of cocoa has extensively investigated in vivo (Mukherjee et al. 2001; Kurosawa et al. 2005; Sies et al. 2005; Ono et al. 2003). To the best of our knowledge, few studies have investigated the effect of the cocoa polyphenolic extract (CPE) on PGE_2_ and 5-LOX and the available information on this effect is limited. To close this gap, the aims of the current study were to (a) determine the influence of CPE on 5-LOX and PGE_2_ and (b) investigate the effect of CPE on the production of ROS, TNF-α and NO. To illustrate the mechanism of action of CPE, LPS-sensitized RAW 264.7 macrophages were used to analyze the production of ROS, NO, PGE_2_, TNF-α and 5-LOX using a synthetic substrate (soybean lipoxygenase).

## Methods

### Preparation of cocoa polyphenolic extracts (CPE)

Malaysian cocoa powder was kindly gifted by KL-Kepong Cocoa Products Sdn. Bhd. (Port Klang, Selangor, Malaysia). The cocoa extract was prepared following the method described by (Ruzaidi et al. (2005). Briefly, the defatted powder was treated with 80 % (v/v) ethanol for 2 h. The ethanol was removed using a rotary evaporator (Buchi Rotavor R-200, Flawil, Switzerland) at 55 °C for 45 min. The resulting extract was lyophilized through freeze-drying (The Virtis Company Inc., Gardiner, NY, USA) at 45 °C and 120 bar.

### Phenol and flavonoid contents CPE

The total amounts of phenols and flavonoids were measured following the method described by Schinella et al. 2010. The total phenol content was determined using the Folin–Ciocalteu reagent and gallic acid as the standard and is expressed as mg of gallic acid equivalent (GAE)/100 ml of extract. The total flavonoid content was measured in a 10 % AlCl_3_·3H_2_O solution using (+)-catechin as the standard and is expressed as mg of catechin equivalent (CE)/100 ml of extract.

### Cell culture

The murine monocytic macrophage-like cell line RAW 264.7 from the American Type Cell Culture Collection (Manassas, VA, USA) was cultured in Dulbecco’s modified Eagle’s medium (DMEM) supplemented with 2 mM glutamine, 100 units/ml penicillin, 100 µg/ml streptomycin, 10 mM 4-[2-hydroxyethyl]-1-piperazineethanesulfonic acid (HEPES) and 10 % fetal bovine serum (FBS) and incubated at 37 °C in a 5 % CO_2_ atmosphere. After reaching 80–90 % confluence, the RAW 264.7 cells were removed, trypsinized and centrifuged at 120×*g* and 4 °C for 10 min. The cells were then treated with serial concentrations of CPE from 15.63 to 1000 µg/ml and 10 µg/ml lipopolysaccharide (LPS).

### Cell viability by MTT assay

The cytotoxicity of CPE on seeded RAW 264.7 cells was evaluated by measuring the formation of formazan salts due to the reduction of 3-(4,5-dimethylthiazol-2-yl)-2,5-diphenyl tetrazolium bromide (MTT). The cells were cultured for 18 h and then treated with LPS (10 µg/ml) and serial concentrations of CPE for 24 h. Then, 20 µl of 5 mg/ml MTT was added to each well and the wells were incubated for 4 h at 37 °C. The formazan crystals were then dissolved by the addition of 200 µl of dimethyl sulfoxide (DMSO) at 37 °C for 30 min. The optical density of the wells was read at 570 nm using a microplate reader (Molecular Devices Inc., Sunnyvale, CA, USA). The rate of cell death was specified relative to that of the control group.

### Quantification of reactive oxygen species

The ROS formation was quantified by measuring the conversion of 2′7′-dichlorofluresceine diacetate (DCFH-DA) to dichlorofluoresceine (DCF) through ROS oxidation (LeBel et al. 1990). At 80 % confluence, RAW macrophage cells were treated with serial concentrations of CPE and 10 µM H_2_O_2_ for 24 h. The treated cells were washed with PBS and incubated with 2′, 7′-dichlorofluorescein diacetate (DCFH-DA) for 15 min in the dark at 37 °C. After washing, the cells were lysed in buffer (50 mM Tris–HCl, 100 mM NaCl, 1 mM CaCl_2_, 1 mM MgCl_2_, 300 mM sucrose, 1 % Triton X-100, pH 7.4) and the fluorescence of the lysates was measured at 529 nm with an excitation wavelength of 495 nm in a stirred quartz cuvette. The DCF fluorescence density is proportional to the amount of intracellular ROS.

### Quantification of nitric oxide (NO)

The RAW 264.7 cells were cultured in 96-well plates (1 × 10^6^ cells/100 ml) for 24 h at 37 °C in a 5 % CO_2_ atmosphere. Then, 1000 μg/ml CPE was diluted and added to the well to obtain final concentrations of 500, 250, 125, 62.5, 31 and 15.6 μg/ml. The cells were then sensitized with 200 U/ml interferon-gamma (IFN-γ) and 10 μg/ml LPS for 20 h. The quantity of nitrite, which is a steadily oxidized product of NO, was measured in tissue culture media using the Griess reagent [1 % (w/v) sulfonamide and 0.1 % (w/v) *N*-(1-naphtyl)ethylenediamine dihydrochloride in 2.5 % (v/v) phosphoric acid]. To summarize, 100 ml of the cell culture fluid was combined with 100 µl of the Griess reagent in a 96-well plate and the absorbance was then read spectrophotometrically using a microplate reader at 540 nm. Dilutions of sodium nitrite were used to obtain a standard curve to determine the amount of nitrite in each sample (Di et al. 1996).

### Quantification of lipoxygenase

CPE at different concentrations ranging from 1000 to 15.625 µg/ml was prepared in DMSO. Sodium phosphate buffer (160 μl, 0.05 M, pH 7.5), 10 μl of the test solution and 20 μl of linoleic acid solution were mixed and incubated for 10 min at 25 °C. The reaction was then initiated by the addition of 10 μl of the substrate in the form of soybean lipoxygenase solution. The enzymatic conversion of sodium linoleic acid to (9Z,11E)- (13S)-13-hydroperoxyoctadeca-9,11-dienoate was measured by monitoring the change in the absorbance at 295 nm over a period of 6 min using a spectrophotometer. Nordihydroguaiaretic acid (NDGA) was used as the positive control in this assay. All of the tests were performed in triplicate in a 96-well UV microplate (Frum and Viljoen 2006).

### PGE_2_ and TNF-α assay

The PGE_2_ and TNF-α level in the RAW macrophage culture medium were quantified using ELISA kits according to the manufacturer’s instructions (Sigma-Aldrich). The production of PGE_2_ and TNF-α was measured relative to that of the control. All of the experiments were performed in triplicate.

### Data analysis

The values are presented as the means of three replicate determinations ± the standard error of the mean (±SEM). All of the data were subjected to one-way analysis of variance (ANOVA) to test whether there are significant differences in the anti-inflammatory activity of CPE and the significance of the difference between the means was determined using Duncan’s multiple-range test (*p* < 0.05). The data analyses were performed using SPSS for Windows (version 18.0).

## Results

### Phenol and flavonoid contents

The total phenols and flavonoid contents in the CPE are presented in Table 1. The concentration of phenolic acids (114 mg/g) in the CPE was higher than the flavonoid concentration (94.95 mg/g).

**Table 1:** Contents of flavonoids and phenolic acids in CPE

Phenolic acid amount	114 mg/g
Flavonoid amount	94 mg/g
Yield extract^a^	23 g/100 g

^a^Yield (percent) = [solvent extracts wt (g)/sample wt (g)] × 100

### Viability of RAW 264.7 cells

**Fig. 1:**
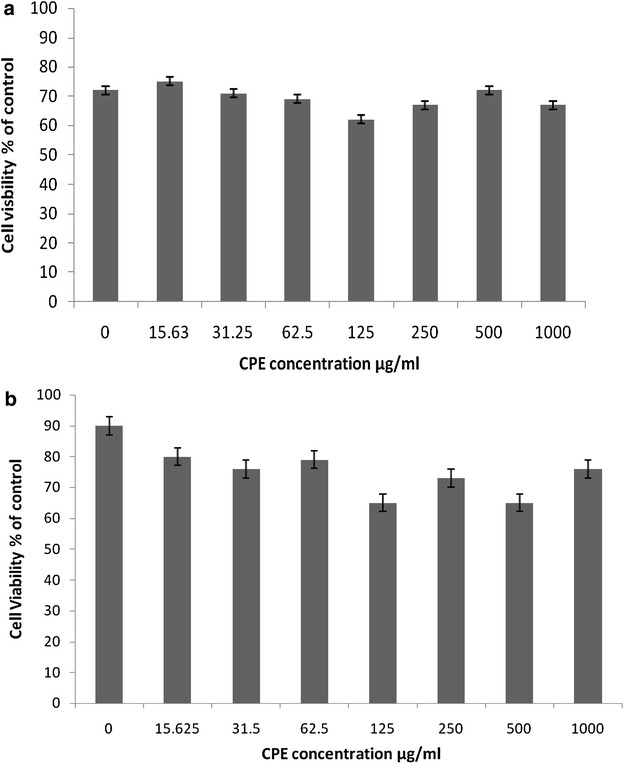
**a** Cell viability of cells treated with various concentrations of CPE and LPS for 24 h. The cell viability was determined by the MTT assay as described in “Methods” section. The results are expressed as the mean ± SEM (n = 3). **b** Cell viability of cells treated with various concentrations of CPE only for 24 h. The cell viability was determined by the MTT assay as described in in “Methods” section. The results are expressed as the mean ± SEM (n = 3)

To confirm the nontoxic effect of CPE on RAW 264.7 cells, the viability of the cells was examined using the MTT assay. As shown in Fig. 1, the treatment of LPS-stimulated cells with CPE at a concentration up to 1000 μg/ml did not affect the viability of the cells compared with untreated LPS-stimulated cells. The highest inhibition rate was approximately 20 %.

### Inhibitory effect of CPE on ROS formation

**Fig. 2:**
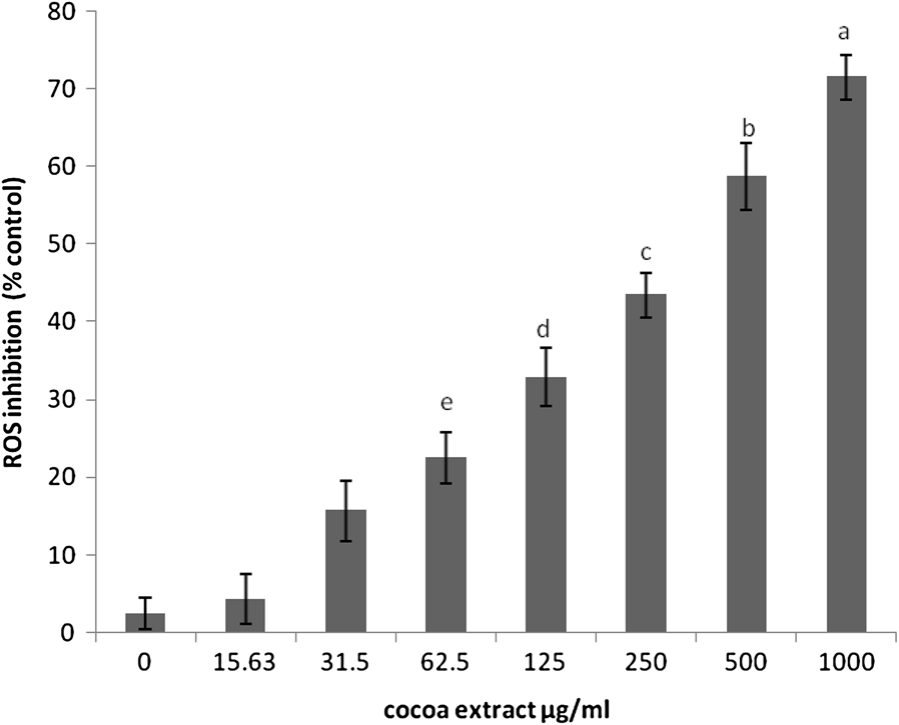
Inhibition of ROS in RAW 264.7 cell cultures treated with CPE for 24 h. The results are expressed as the mean ± SEM (n = 3). The means with *different letters* were significantly different (*p* < 0.01)

To investigate whether CPE can inhibit intracellular ROS, RAW 264.7 macrophage cells were treated with varying concentrations of CPE and then exposed to H_2_O_2_. A fluorescence protocol based on 2′7′-dichlorofluresceine diacetate (DCFH-DA) was used to measure the intracellular ROS. CPE significantly (*p* < 0.01) suppressed the H_2_O_2_-induced intracellular ROS accumulation in a dose-dependent manner compared with the untreated cells (Fig. 2).

### Inhibitory effect of CPE on Nitric oxide

**Fig. 3:**
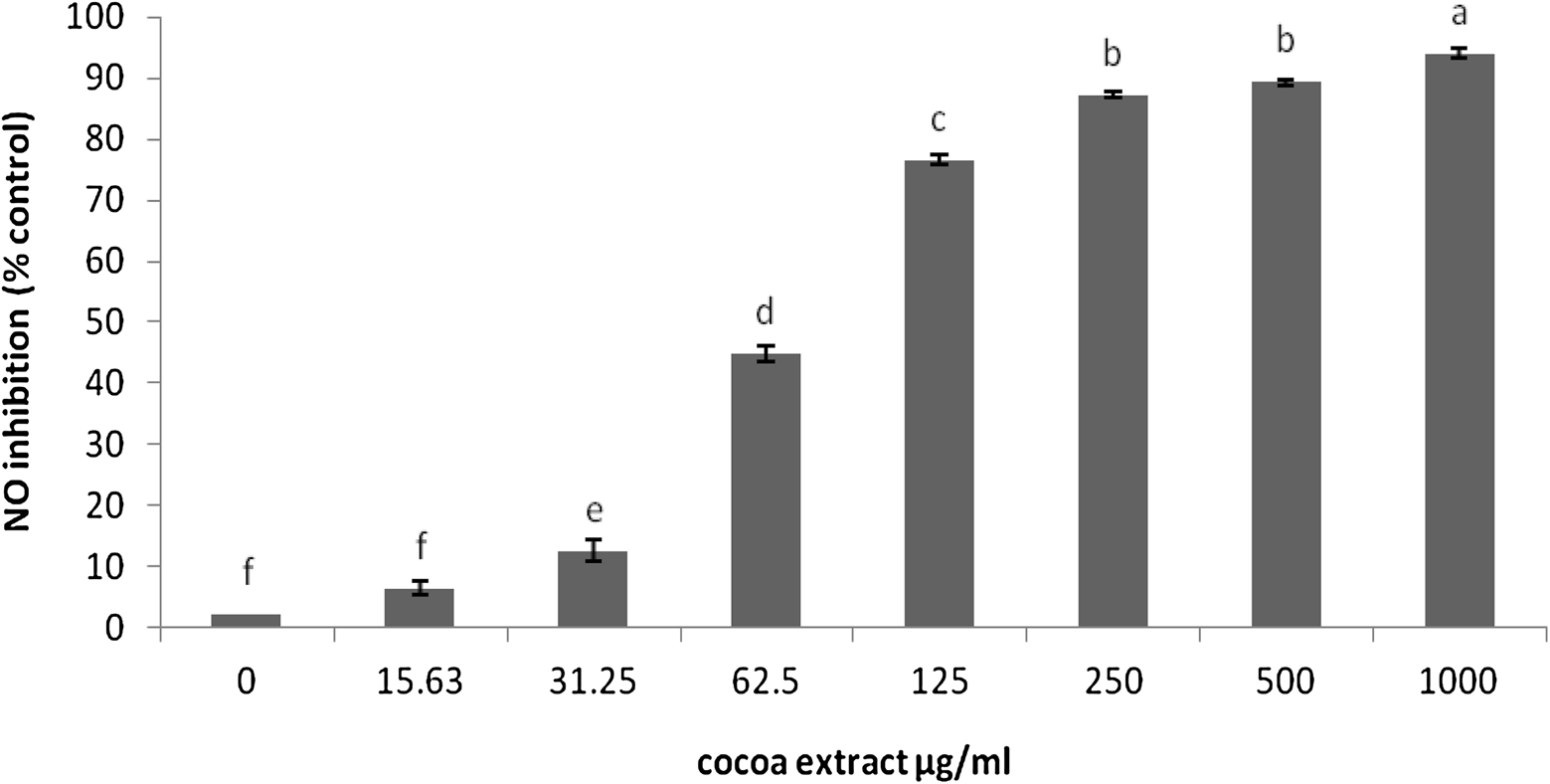
The cells were treated with serial concentrations of CPE for 20 h and then with 200 U/ml IFN-γ and 10 μg/ml LPS. The results are expressed as the mean ± SEM (n = 3). The means with *different letters* were significantly different (*p* < 0.01)

As described below, LPS stimulation caused a significant production of NO in the culture medium. However, the pretreatment of the cells with CPE at different concentrations significantly inhibited the LPS-induced nitrite accumulation in a dose-dependent manner (Fig. 3).

### Inhibitory effect of CPE on 5-lipoxygenase

**Fig. 4:**
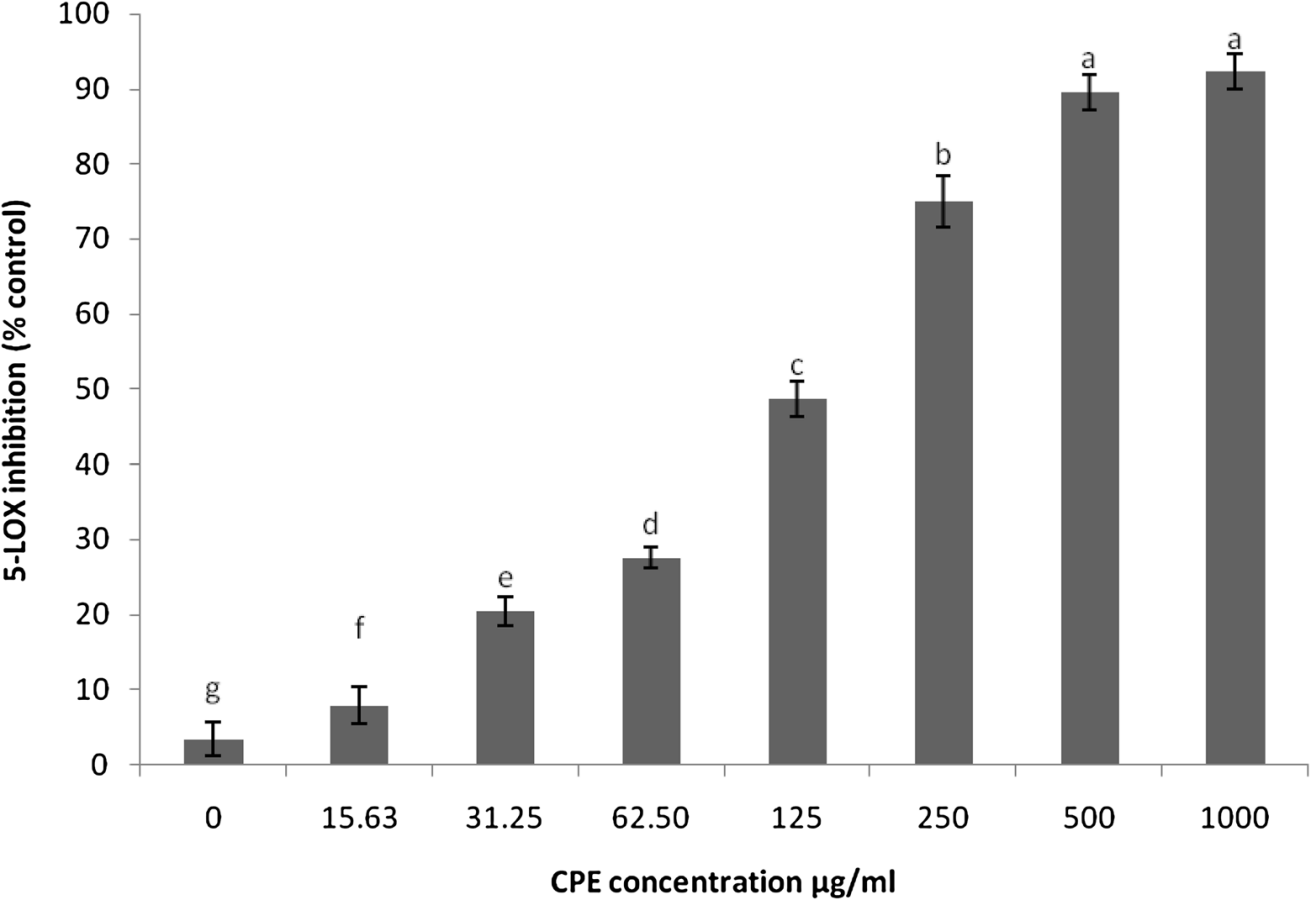
Inhibition of soybean 5-LOX by CPE. The results are expressed as the mean ± SEM (n = 3). The means with *different letters* were significantly different (*p* < 0.01)

**Fig. 5:**
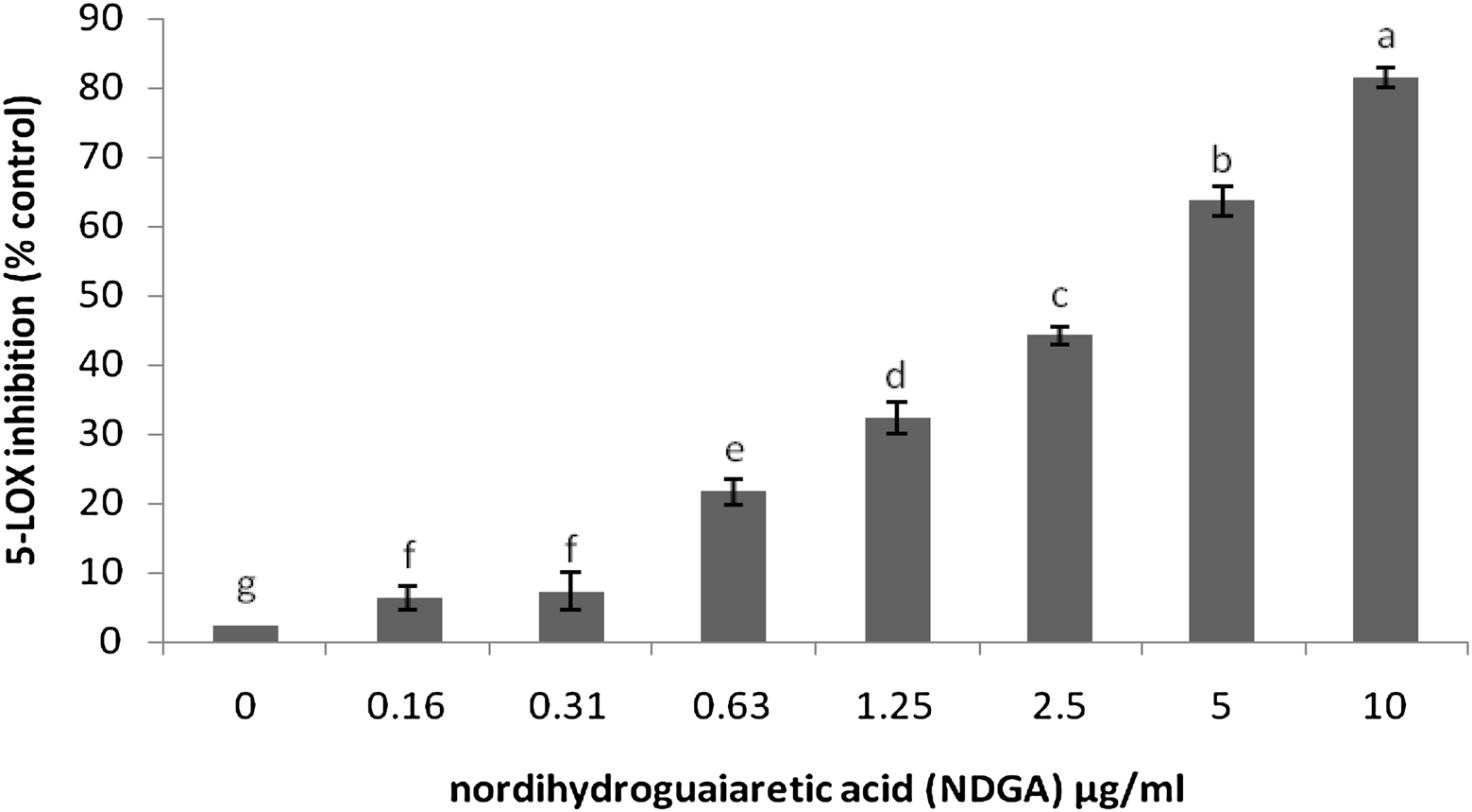
Inhibition of soybean 5-LOX by NDGA as a positive control. The results are expressed as the mean ± SEM (n = 3). The means with *different letters* were significantly different (*p* < 0.01)

Similarly to previous studies on purified enzymes, CPE was found to potently affect the activity of purified 5-LOX. The enzyme inhibition by CPE was concentration-dependent at concentrations ranging from 15.63 to 1000 µg/ml with an IC_50_ value of 155 µg/ml (Fig. 4). NDGA, which was used as a positive control, gave an IC_50_ value of 4 µg/ml (Fig. 5).

### Inhibitory effect of CPE on LPS-induced PGE_2_ and TNF-α production

Because PGE_2_ and TNF-α are inflammatory mediators, the effects of CPE on PGE_2_ and TNF-α production in LPS-stimulated RAW 264.7 cells were also measured (Table 2). The incubation of the cells with serial concentrations of CPE prior to LPS treatment caused a significant dose-dependent decrease (*p* < 0.05 or *p* < 0.01) in the production of PGE_2_ and

**Table 2:** Effect of CPE on the inhibition of pro-inflammatory mediators in RAW 246.7 macrophages

Inflammatory mediators	IC_50_ (µg/ml)
ROS	425.95
NO	81.59
5-LOX	166.48
TNF-α	52.28
PGE_2_	27.57

### TNF-α compared with the untreated LPS-stimulated cells (Figs. 6, 7)

## Discussion

Natural products have played a significant role not only in clinical nutrition against several diseases but also in drug discovery and development by contributing to the discovery of alternative therapies. In addition, the active participation of macrophages in the inflammatory response by the secretion of mediators induced by pathogenic-derived factors, such as LPS and IFN-γ, contributes to the establishment of in vitro inflammatory models (Zhang et al. 2010; Yoon et al. 2009). In the current study, the ethanolic polyphenol extract from cocoa (CPE) was prepared and its effects on the LPS-induced inflammation in a murine macrophage cell line (RAW 264.7) were examined. The cytotoxicity of CPE in RAW 264.7 cells was also assessed using the MTT assay. The findings showed that CPE does not influence the viability of RAW 264.7 cells.

**Fig. 6:**
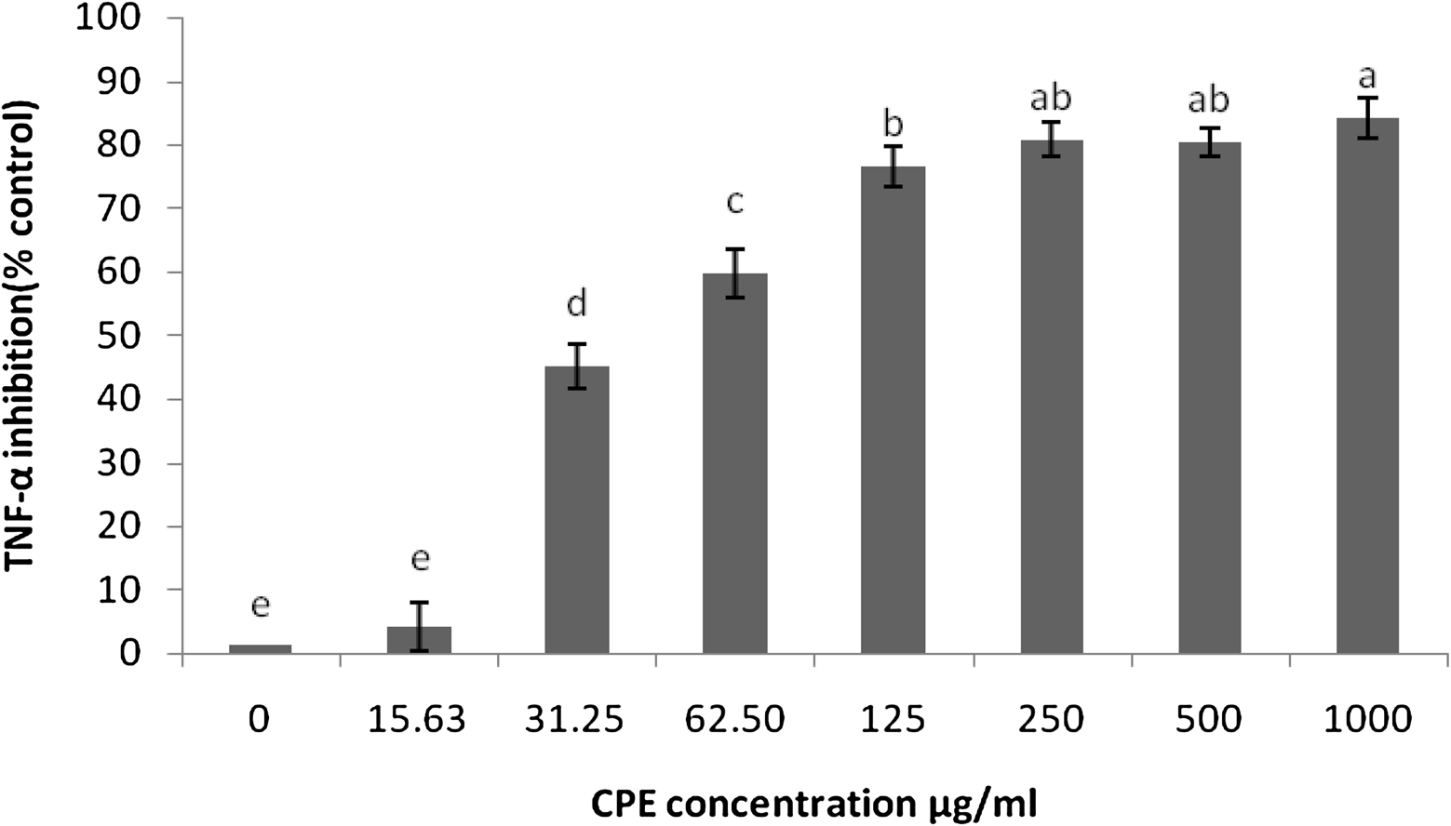
Effects of CPE on the LPS-stimulated production of inflammatory mediators in RAW 264.7 cells. The TNF-α inhibition was determined using an ELISA kit. The results are expressed as the mean ± SEM (n = 3). The means with *different letters* were significantly different (p < 0.01 or p < 0.05)

**Fig. 7:**
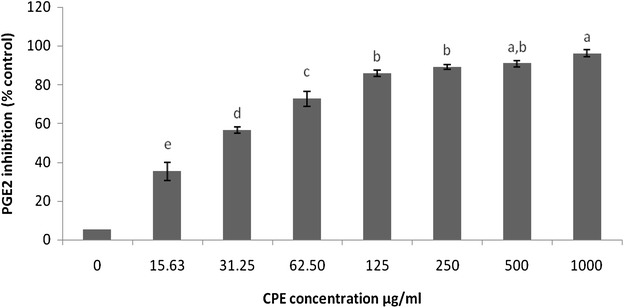
Effects of CPE on the LPS-stimulated production of inflammatory mediators in RAW 264.7 cells. The PGE_2_ inhibition was determined using an ELISA kit. The results are expressed as the mean ± SEM (n = 3). The means with *different letters* were significantly different (p < 0.01 or p < 0.05)

An active stimulant to inflammation is ROS and it has been noted that the ability of macrophages to produce ROS depends on the level of tissue damage. It is noteworthy that the chemotactic factors released from inflammatory cells may accumulate and induce the release of ROS. In the current study, CPE was found to significantly inhibit ROS with an IC_50_ value of 425.95 µg/ml. These results are consistent with those reported by Rodriguez-Ramiro et al. (2011) who showed the inhibition of ROS production in the Caco-2 cell line by procyanidin B2 and CPE. In contrast, Chai et al. (2003) showed the production of a high concentration of ROS in cell cultures incubated with epigallocatechin gallate (EGCG) derived from wine and green tea.

Of the many inflammatory mediators that can stimulate vascular permeability, it is well documented that NO, PGE_2_ and LT are the primary factors implicated in the pathogenic process of several inflammatory diseases (Salmon and Higgs 2012; Bi et al. 2005), iNOS, which is expressed and activated in different cell types through stimulation with TNF-α and/or LPS, has the ability to increase the NO concentration (Mac Micking et al. 1997). Under specific conditions, the treatment of stimulated RAW 264.7 cells with CPE results in a considerable inhibition of NO production. However, the mechanism responsible for the downregulation of NO production by polyphenols and flavonoids has not been fully elucidated. It has been reported that this effect is most likely due to the integration of multiple different bioactivities, such as the suppression of the activity of the iNOS enzyme, the scavenging activity of NO and the suppression of the mRNA expression of iNOS (Moncada et al. 1991). In addition, TNF-α is released in small quantities under normal condition, but this amount increases during the inflammatory state stimulated by LPS in macrophages. Cells treated with different concentrations of CPE showed a significant inhibition of TNF-α production (Fig. 6; Table 1). Moreover, the TNF-α-NO pathway has been found to be inhibited by iNOS inhibitors (corticosteroids) (Wolf et al. 2005). Similarly, Bi et al. (2005) and Park et al. (2000) also reported that flavonoids and resveratrol exert inhibitory effects on NO and TNF-α in an in vitro model.

More importantly, the repression of the biogenesis of inflammatory mediators, particularly PGE_2_ and LT, is considered a promising method for the management of different types of diseases associated with inflammation, such as osteoarthritis (Celotti and Laufer 2001). Recent studies discuss ‘dual inhibitors’, which are agents that have the capability to suppress not only COX-1 and COX-2 but also 5-LOX (Brune 2004). Our experiments revealed the inhibitory activity of CPE on the production of PGE_2_ and 5-LOX (Figs. 4, 5; Table 1) by targeting the COX-PGE_2_ and 5-LOX-LT pathways. Consistent with the findings reported by Altavilla et al. (2009) we found that flavonoids have a suppressive effect on the production of PGE_2_ and LT. Additionally, sinapic acid, one of the polyphenol components, has been shown to inhibit COX-2 in RAW macrophage cells (Yun et al. 2008). It should be noted that, the different polyphenols exert different effects on pro-inflammatory mediators; some polyphenols suppress pro-inflammatory mediators whereas others can induce the production of these mediators. Surprisingly, the highest IC_50_ value found for these mediators was related to ROS and this value suggested a suitable inhibitory concentration.

## Conclusion

Our results support the findings of previous reports on the anti-inflammatory activity of cocoa and its by-products. However, a direct comparison cannot be established with other compounds mentioned in the literature due to differences in the experimental assays. The current results strengthen the underlying evidence of the protective effect of CPE against the initiation of the inflammatory process associated with several diseases. Furthermore, CPE markedly attenuated the tested inflammatory signs by blocking the TNF-α-NO, COX-II-PGE_2_ and 5-LOX-LT pathways. These findings provide a rationale for the application of CPE in clinical nutrition for the treatment of chronic diseases. In addition, the findings obtained in our in vitro study and other studies on human subjects and laboratory animals suggest that the improvement of the inflammatory state is a pivotal action of the dietary polyphenols derived from cocoa. However, further in vivo and in vitro studies, as well as human intervention trials, are required to clarify the mechanism through which cocoa polyphenols can prevent inflammation.

## Abbreviations

CPE: cocoa polyphenolic extract
COX-2: cyclooxygenase-2
IFN-γ: interferon-gamma
IL-1β: interlukin-1β
IL-6: interlukin-6
iNOS: nitric oxide synthase
LPS: lipopolysaccharide
5-LOX: 5-lipoxygenase
MTT: 3-(4,5-dimethylthiazol-2-yl)-2,5-diphenyl tetrazolium bromide
NO: nitric oxide
PGE_2_: prostaglandin E2
ROS: reactive oxygen specious
TNF-α: tumor necrosis factor-alpha
